# Awareness of health effects of cooking smoke among women in the Gondar Region of Ethiopia: a pilot survey

**DOI:** 10.1186/1472-698X-8-10

**Published:** 2008-07-18

**Authors:** M Edelstein, E Pitchforth, G Asres, M Silverman, N Kulkarni

**Affiliations:** 1 Hemel Hemspead General Hospital, Hillfield Road, Hemel Hemspead, UK; 2 Department of Health Sciences, University of Leicester, UK; 3 Gondar College of Medical Sciences, Gondar, Ethiopia; 4 Department of Infection, Immunity and Inflammation, Division of Child Health, University of Leicester, UK

## Abstract

**Background:**

The burning of biomass fuels results in exposure to high levels of indoor air pollution, with consequent health effects. Possible interventions to reduce the exposure include changing cooking practices and introduction of smoke-free stoves supported by health education. Social, cultural and financial constraints are major challenges to implementation and success of interventions. The objective of this study is to determine awareness of women in Gondar, Ethiopia to the harmful health effects of cooking smoke and to assess their willingness to change cooking practices.

**Methods:**

We used a single, administered questionnaire which included questions on household circumstances, general health, awareness of health impact of cooking smoke and willingness to change. We interviewed 15 women from each of rural, urban-traditional and middle class backgrounds.

**Results:**

Eighty percent of rural women cooked indoors using biomass fuel with no ventilation. Rural women reported two to three times more respiratory disease in their children and in themselves compared to the other two groups. Although aware of the negative effect of smoke on their own health, only 20% of participants realised it caused problems in children, and 13% thought it was a cause for concern. Once aware of adverse effects, women were willing to change cooking practices but were unable to afford cleaner fuels or improved stoves.

**Conclusion:**

Increasing the awareness of the health-effects of indoor biomass cooking smoke may be the first step in implementing a programme to reduce exposure.

## Background

It is estimated that around 3 billion people worldwide rely on wood, stubble, dung and leaves for cooking fuel. Burning biomass fuels on open fires and in inefficient stoves, releases many harmful pollutants [1]. Inhaling these pollutants results in excess respiratory morbidity and mortality in women and children [2]. Known as a "silent killer" [1], over 1.6 million children die annually throughout the developing world from the consequences of exposure to biomass fuel smoke [3]. Exposure to indoor air pollution has been linked with i) a reduction of forced vital capacity [4] ii) an increased risk of acute respiratory infections [5] iii)chronic obstructive pulmonary disease (COPD)[6] and iv) lung cancer [7] in women. We have shown higher amounts of carbon in sputum macrophages in women and children exposed to biomass smoke in Ethiopia as compared to those using cleaner fuels in England, suggesting higher amount of pulmonary exposure [8]. Exposure to biomass smoke is therefore a major public health issue [9].

Ethiopia, a country of 75 million people in East Africa, is one of the poorest countries in the world, where 80% of the population lives in a rural setting [10]. The use of biomass fuel as primary source of cooking fuel is widespread throughout the country. Use of improved stoves in households reduces levels of indoor air pollution [11]. Policy interventions to change cooking methods have been shown to be effective in reducing morbidity and mortality associated with indoor air pollution [12] Such interventions may vary from practical measures such as cooking outside, to installation of improved stove designs. Wide ranges of improved stove models exist, varying in cost, technology and efficiency. These stoves are designed to be affordable to target populations and rely on locally available fuel such as dung or wood [13]. Uptake and acceptance of new cooking methods or stoves is vital to the success of any policy intervention. This said, data on awareness of problems associated with exposure to indoor air pollution is lacking [14]. Further potential barriers to change include social and cultural barriers [15] and cost. Other factors contributing to poor acceptance include i) lack of training in new cooking methods, ii) poor quality and inadequate maintenance and iii) improper design of stoves [16].

Before attempting to intervene to reduce the health impacts of biomass cooking smoke, it is essential to assess awareness of the impact of indoor air pollution on health and willingness to change. This is, to our knowledge the first study addressing these issues.

## Methods

### Setting and Study population

The study was based in Gondar, a city of 200,000 inhabitants. It is the third largest city in Ethiopia and one of the major cities of the state of Amhara, located 800 km from the capital Addis Ababa. It is essentially a rural area, with 88.5% of the population estimated to be rural inhabitants, the majority of whom use biomass as a main cooking fuel. There is a mix of lower and middle socio-econnomic class families living within the community. The city is surrounded by a multitude of small villages from which data for rural dwellings was obtained. The population speaks Amharic and the level of illiteracy is high in rural areas. Smoking is rare amongst Ethiopian women especially outside major urban centres. The target population for our study was women as they have primary responsibility for cooking in the household. Women were recruited from Gondar city and the surrounding rural areas.

### Sampling

Women were recruited by household depending on type of dwelling (rural, traditional urban and middle class) in order to include women of different socioeconomic status. Approval for the study was obtained from local ethics committee in Gondar. Participants from non-smoking families were recruited by walking from house to house and asking for consent to be part of the study. For our pilot study, a target sample of forty-five women, fifteen in each group, was set. Rural households were sampled from villages 5 km outside Woreta, a small town 120 km from Gondar. All rural households were situated at least 1 km from the main road. These houses were built out of mud and wood and did not have electricity or access to piped water. All participants in these villages were from subsistence farming households, representing the majority of the households in Ethiopia. 'Traditional urban' households were sampled from within the city of Gondar. All had electricity and a source of piped water close by. Houses built out of concrete (indicator of higher socio-economic status) were not eligible. Middle class households were sampled from a professional, residential area of Gondar called the American Village. Houses had to be built from concrete to qualify. Although smoking was an exclusion criterion, we did not meet any female smokers during the study.

### Questionnaire design

A questionnaire was designed specifically for the purpose of this study (see Additional file 1). Questions were divided into four sections; household circumstances, general health, awareness of cooking smoke causing illness and willingness to change cooking practices. Questions around household circumstances were derived from the Ethiopian rural household survey [17]. These questions related to the size of the house, access to domestic facilities such as water or electricity, type of fuel most commonly used, profession of the husband or total house income. The remaining questions were informed by a literature review and expertise of the research team.

In terms of reported health, we asked closed questions first to establish whether women linked indoor air pollution and disease. We then asked women to elaborate using open-ended questions. Preliminary questions were asked about general health before focusing on respiratory health. The open-ended questions were designed to allow women to describe their health problems in their own terms. We assessed awareness of the problems caused by indoor cooking fume by asking whether the interviewees thought cooking smoke was detrimental to their health. They were then asked to elaborate, again using open-ended questions.

The last section started with a statement assuring women that indoor air pollution was detrimental to their health and was assessing their behaviour based on this assumption. The women interviewed did not question the authority as the statement was coming from medical doctors. Women were asked whether they would be willing to change their habits based on this new knowledge and if so, how. We also asked womens opinions about the quality of different fuels.

Written initially in English, the questionnaire was translated into Amharic, and independently back translated for verification. Two medical doctors, one English speaking and one bilingual, trained in data collection, administered the questionnaire. The Amharic-speaking interviewer asked all questions in Amharic and translated the answers into English immediately. Open-ended responses were recorded word for word.

### Analysis

Questionnaire responses were entered into SPSS (SPSS version12.0.1); checked and descriptive analysis was undertaken. Content analysis was used to analyse the open-ended responses. This involved reading and re-reading responses to the same questions, identifying recurring themes and categorising all responses to a particular theme to allow numerical analysis.

## Results

Forty-five women, fifteen from each sub-group, completed the questionnaire. No women refused to participate or had to be excluded because of smoking. The household characteristics of the three groups are shown in Table 1.

**Table 1:** Household characteristics by socio-economic group

	**Rural (n = 15)**	**Traditional-urban (n = 15)**	**Middle Class (n = 15)**
**Age (yr) **– mean (SD)	34.3 (14.4)	38.6 (11.9)	32.6 (13.6)
Women's education			
Yes	0	6	12
Completed schooling (12^th ^grade)	0	0	5
**Number of children **Median (Range)	3 (0–8)	3(0–8)	3 (0–7)
**No of rooms **Median (Range)	1 (1–1)	1 (1–2)	2 (1–5)
**Source of drinking water -No (%)**			
Tap inside compound	0	6 (40)	9 (60)
Tap inside house	0	0	6 (40)
Tap outside compound	0	9 (60)	0
Open well	15 (100%)	0	0
**Salary -Birr/month **Median (Range)*	0 (0–50)	1900 (800–10000)	300 (100–1500)

* 9.21 Birr = 1 US Dollar

### Household circumstances

All the men in rural households were self-sustaining farmers, typically farming on a small plot of land in order to provide for their families. They would generally not use money on a regular basis, and had no income as they rarely sold their produce. Their houses typically comprised of one living space where all the cooking was done. They had no access to water, electricity, sanitation or education (Table 1). The women typically travelled several miles to fetch water. By contrast, middle class head of households held Social class II or I jobs such as teachers, doctors or civil servants. The houses were well equipped and bigger, with universal access to water, electricity and a kitchen. The women were better educated. Urban traditional dwellings fell between and were less homogenous than the two other groups, with access to electricity and to water within the compound in 40% of cases. These socio-economic differences were not reflected in the time spent cooking (3 hours per day median, range 1–10).

### Cooking practices

Disparities were reflected in cooking practices across the different socioeconomic groups (Table 2). Middle class households had adequate cooking circumstances, with 87% cooking in a well ventilated, designated area, mostly with wood or charcoal, which is perceived as a "clean" fuel, and is more popular than electricity. By contrast, the majority of rural families (80%) cooked inside, using dung, with no ventilation. Two thirds of traditional urban households cooked outside, and a quarter had a well-ventilated, separate area to cook.

**Table 2:** Cooking practices amongst Ethiopian households

	**Rural (n = 15)**	**Traditional (Urban) (n = 15)**	**Middle class (n = 15)**
**Cooking area: **no (%)			
Within living area	12 (80)	1 (6.7)	0
Outside	1 (6.7)	10 (66.7)	2 (13.3)
Separate area	1 (6.7)	0	4 (26.7)
Separate room	1 (6.7)	4 (26.7)	9 (60)
**Cooking Fuel Predominantly used: **no (%)			
Charcoal	0	6 (40)	7 (47)*
Cow dung	14 (93.3)	1 (6.7)	0
Electricity	0	0	4 (26.7)
Sawdust	0	2 (13.3)	0
Wood	1 (6.7)	6 (40)	4 (26.7)
**Ventilation: no (%)**			
Cooks outside	1 (6.7)	10 (66.7)	1 (6.7)
Electricity	0	0	4 (26.7)
No ventilation	11 (73.3)	1 (6.7)	1 (6.7)
Poor ventilation	0	1 (6.7)	1 (6.7)
Average ventilation^†^	2 (13.3)	0	0
Well ventilated^‡^	1 (6.7)	3 (20)	8 (53.3)

*Used electricity as well

^† ^Ventilation through a gap between the top of the walls and the roof.

^‡ ^Special efforts made to improve ventilation i.e. cooking in a specifically designed shed with no walls.

### General health status

Women from rural and traditional backgrounds reported more ill health than middle class women. While only 33% of women from middle class backgrounds reported ill health, the figures were 73% and 80% for rural and urban traditional women, respectively. Women from rural and traditional households also reported more ill health in their children (33% in each) than women from middle class households (7%). A similar trend was shown when asked specifically asked about respiratory problems (Table 3). The respiratory symptoms/diagnosis reported were breathing discomfort (6 women), cough (4 women), asthma (1 woman) and shortness of breath (5 women). The symptoms/diagnosis reported in children were chest pain (1 child), cough (1 child) and asthma (1 child).

**Table 3:** Respiratory health of women and their children

**Respiratory health problems**	**Rural (n = 15)**	**Traditional-Urban (n = 15)**	**Middle class (n = 15)**
**Mother -Number (%)**			
No	8 (53.3)	11 (73.3)	13 (86.7)
Yes	7 (46.7)	4 (26.7)	2 (13.3)
**Children- Number (%)**			
No	12 (80)	14 (93.3)	12 (80.0)*
Yes	2 (13.3)	1 (6.7)	0

*One rural woman had no children; 3 middle class mothers had no children

### Awareness of adverse health effects

The vast majority of women across social groups were aware that cooking fumes could cause ill health, although it was recognised more often as a cause of illness in women than in children, especially amongst rural families (Table 4). Only a small percentage of women in rural households identified cooking smoke as a factor of respiratory disease in children, and an even smaller percentage were concerned about it. Women associated smoke more commonly with eye disease, TB or throat problems (19 women) than cough or other respiratory problems (7 women). Seven women thought indoor cooking smoke caused respiratory problems in children, two linked it with eye disease, and six associated smoke with tiredness and general fatigue without being more specific.

**Table 4:** Women's awareness of the effects of indoor cooking smoke on health

**Awareness of adverse affects of biomass smoke**	**Number aware/not aware or not concerned**		
	**Rural (n = 15)**	**Traditional urban (n = 15)**	**Middle class (n = 15)**

General health	11/4	12/3	11/4
Breathing problems in children	3/12	10/5	12/3
Breathing problems in mother	10/5	12/3	10/5
Concerned about own health	7/8	10/5	8/7
Concerned about children's health	2/13	9/6	7/8

### Willingness to change fuel and cooking practices

Most women acknowledged that some fuels were better than others (Table 5). Wood and charcoal are viewed positively in the target group, even amongst middle class households. Traditional urban and middle class families, who in many cases do not use it even though they have access because of cost, mentioned electricity. 90% of women indicated they would be willing to change fuel if they were told it could improve their health or the health of their children. However cost was a major issue. The majority of poorer households (93%) could not afford any contribution to an improved system, compared to 33% of middle class households (Table 5). Fifty three percent of traditional urban families could only afford 5 extra Birr/week. No rural families could afford any contribution to a smoke free stove but 40% of traditional urban families were willing to spend an average of 125 Birr (range 30–300) for a new stove, and 65% of middle class families would spend 2000 Birr (range 300–5000). All women were ready to cook outside or in a separate area if it would improve their health, although some women mentioned that bad weather such as rain, sun or wind prevented them from cooking outside. All but one woman would welcome a free smoke-free system in their house.

**Table 5:** Awareness of cleaner fuels and willingness to change

**Willing to change**	**Rural (n = 15)**	**Traditional-Urban (n = 15)**	**Middle class (n = 11)**
**Are some fuels better?**			
Don't know	1 (6.7)	1 (6.7)	0
No	2 (13.3)	2 (13.3)	0
Yes	12 (80)	12 (80)	11*
			
**Which fuel is best?**			
Charcoal	3 (20.7)	4 (26.7)	3 (27.3)
Charcoal and electricity	0	0	1 (9.1)
Charcoal and wood	2 (13.3)	0	1 (9.1)
Charcoal, wood, gas	0	1 (6.7)	0
Cow dung and wood	2 (13.3)	0	0
Electricity	0	4 (26.7)	4 (36.4)
Gas	0	0	0
Wood	5 (33.3)	3 (20)	2 (18.2)
			
**Extra sum willing to spend Birr (/week) on cleaner fuel Median (range) **^†^	0	5 (4–25)	12.5 (10–37.5)

* Four women used electricity

^† ^9.21 Birr = 1 US Dollar

## Discussion

This pilot study is the first to evaluate women's awareness of health issues related to indoor air pollution and the harmful effects of cooking smoke. It is also the first to assess women's willingness to change cooking practices and the possible impact of health education on it. Ethiopian society is characterised by extremes of household income and exposure to indoor air pollution. The conditions of cooking in rural areas (representing about 80% of the country's population) are poor, with women generally cooking indoors in non-ventilated areas, with generally poor living conditions, no access to clean water, electricity or sewage and living, cooking and sleeping in the same space. This is also true to a lesser extent in traditional urban areas. Whereas middle class families, living in comfortable houses with access to water, electricity and a reasonable disposable income, generally have good cooking conditions and the ability to cook using better fuels in well ventilated, designated areas. If women across the socio-economic spectrum acknowledge on the whole that biomass smoke has a negative effect on their health, causing breathing difficulties, only half of women acknowledge it is a danger for their children. This dropped to less than 20% in the rural population, the most vulnerable group, in which biomass smoke is not seen by the majority as a cause for concern. The level of health awareness is low in this group, although the women interviewed did consider some fuels as cleaner than others. Paradoxically, in the same rural population the level of reported respiratory disease is two to three times higher than in urban traditional or middle class groups. The group most affected by indoor air pollution is also the one least concerned about it. A lack of knowledge and a lack of concern are associated with reduced willingness to change.

Lack of knowledge has been identified as barrier to change in other fields[18]. For example, in traditional societies, the belief that visits to antenatal care clinics are not mandatory or useful is one of the reasons for poor attendance[19], even though clinic attendance has been shown to significantly reduce the rate of complication during pregnancy. Although no equivalent data has been published in the field of indoor air pollution, lack of awareness and education has been cited as factors in the failure of improved stoves, for example the Indian National Stove programme. Between 1985 and 2002 the Indian Government installed over 30 million improved cooking stoves. However, the majority of users had reverted to traditional cooking methods within two years. Lack of awareness and education was noted to be one of the reasons for the failure [14].

In the population studied, we found that in spite of a lack of concern in the rural population, and in spite of certain beliefs in the positive value of smoke (smoke kills insects) simple advice by a health professional might significantly change attitudes. Over 90% of women who participated were willing to change their cooking practices when informed that smoke is harmful to themselves and their children. Health education to increase awareness of the dangers of indoor air pollution may be an important first step in modifying health behaviours. The WHO has recently prioritised education alongside better fuels and stoves for reducing indoor air pollution [20]. It is therefore essential that in future populations where the level of knowledge about indoor air pollution is low, physical intervention be accompanied by appropriate education to enhance the long-term sustainability of stove programmes.

There is some evidence to support the use of social marketing in health promotion as health education alone may not result in behaviour change [21]. Social marketing, an economics concept created in the 1970s by Kotler and Zaltman [22], is based on the principle that ideas, attitudes and behaviours can be "sold" to people. Manufacturers of cooking stoves and NGOs have used this technique to promote healthier cooking methods. The arguments used to promote dissemination rarely rely on health messages and tend to emphasize benefits in terms of cooking times or house cleanliness. In addition to promoting better stoves, simple measures such as cooking outside, keeping children away smoke and better ventilation could be advocated. Means to change are an issue. The population in our study most at risk were least able to adopt healthier practices and afford alternative means of cooking.

Looking ahead, a larger survey would strengthen our results and provide a better basis for devising a population awareness campaign. There are further considerations from our study design. We focused on the views of women as they had primary responsibility for cooking within the household. It may have been useful to include household men as they are often in charge of the household's finances, and therefore likely to be influential in household decisions to fund new cooking methods. We did not encounter any improved stoves in use in rural or traditional households. This said it might have been useful to know whether any households had heard of different types of stoves, and whether they had been approached by any organisations regarding this matter, rather than only focusing on fuels. We used ventilation and type of fuel used as proxy markers of level of indoor air pollution as it was beyond the scope of this study to measure pollutant levels.

## Conclusion

Our pilot study is an important step in seeking to assess awareness of the affects of cooking fuel on health and willingness to change among women in Gondar region. Increasing the awareness of the health-effects of indoor biomass cooking smoke may be the first step in implementing a programme to reduce exposure. Our findings have direct relevance for policy and practice, supporting implementation of a programme to increase accessibility and use of improved stoves in the area, along with appropriate education. Further research would help ensure an evidence-based approach and post-implementation evaluation of health and awareness would be vital.

## Competing interests

The authors declare that they have no competing interests

## Authors' contributions

ME participated in the study design, travelled to Gondar for data collection and drafted the manuscript; EP performed the data analysis and advised on manuscript; GA organised and participated in data collection and participated in study design; MS conceived the study and participated in its design; NK was the main project supervisor. She participated in performing data analysis, drafting the manuscript and offering guidance in manuscript writing. All authors read and approved the final manuscript.

## Pre-publication history

The pre-publication history for this paper can be accessed here:



## Supplementary Material

Additional file 1 Pilot Study of public knowledge and attitude to the health effects of cooking smoke, by women in Gondar, Ethiopia: questionnaire. Click here for file
