# Supplementary material for: Awareness of health effects of cooking smoke among women in the Gondar Region of Ethiopia: a pilot survey
Source: BMC Int Health Hum Rights. 2008 Jul 18;8:10. doi: 10.1186/1472-698X-8-10 (PMC2491593; doi:10.1186/1472-698X-8-10)
Supplement: Additional file 1 — Pilot Study of public knowledge and attitude to the health effects of cooking smoke, by women in Gondar, Ethiopia: questionnaire. [file 1472-698X-8-10-S1.doc]

**Pilot Study of public knowledge and attitude to the health effects of cooking smoke, by women in Gondar, Ethiopia**

**Questionnaire**

Interviewee age:

**Section 1: Health**

| Yes | No | Don’t know |
| --- | --- | --- |

1. Do you have any health problems?

-if yes, explain

| Yes | No | Don’t know |
| --- | --- | --- |

1. Do your children have any health problems?

- if yes, explain

| Yes | No | Don’t know |
| --- | --- | --- |

1. Do you have any chest problems?

- if yes, specify for each problem: symptoms, frequency and duration.

1. Do your children have any chest problems?

| Yes | No | Don’t know |
| --- | --- | --- |

- - - if yes, specify for each child and problem: symptoms, frequency and duration.

1. If you have chest problems what do you think the causes are?

**Section 2: Public awareness of cooking smoke and health problems.**

1. Do you think cooking smoke affects the health of women or children?

| Yes | No | Don’t know |
| --- | --- | --- |

- if yes, how?

1. Do you think cooking smoke causes:

| Yes | No | Don’t know |
| --- | --- | --- |

a)-breathing (or chest) problems in children?

-if yes, explain:

b)-breathing problems in women who do the cooking?

| Yes | No | Don’t know |
| --- | --- | --- |

-if yes, explain:

1. Are you concerned about the effects of cooking smoke on:

a)-yourself?

| Yes | No | Don’t know |
| --- | --- | --- |

- if yes, explain:

b)-your children?

| Yes | No | Don’t know |
| --- | --- | --- |

- if yes explain

**Section 3: Household circumstances.**

1. Education

| Yes | No | Don’t know |
| --- | --- | --- |

a)- Have you ever had any formal education?

- if yes, what is the highest grade completed?

b)- Are you able to read and write a simple sentence?

| Yes | No | Don’t know |
| --- | --- | --- |

1. Family structure

-How many children have you had?

1. Income

a)-what is your husband’s job?

b)-what is the monthly income in your house? BIRR

1. House circumstances

| Yes | No |
| --- | --- |

a)-Does your household have electricity?

If yes, what is the source? (mains/generator)

b)-How many rooms in your house are used for sleeping?
 (except separate cooking area + toilet/bathroom)

c)-What is the main source of drinking water for members of your household? (tap inside dwelling/inside compound/outside compound, open well/spring, covered well/spring, river, pond/lake/dam, rainwater, other))

| Yes | No | Don’t know |
| --- | --- | --- |

d)- Are you connected to a sewage system?

e)-Where do you mostly cook? (living area/separate room/outdoors)

f)-What type of fuel does your household mainly use for cooking?

(electricity, lpg/ natural gas, biogas, kerosene, charcoal, firewood, straw,

dung, other)

g)-How much time per day do you spend cooking? (estimate hours)

h)-is there a ventilation system in your house?

**Section 4: Implementing change**

1. Do you think some cooking fuels are better for your health than others?

| Yes | No | Don’t know |
| --- | --- | --- |

-If yes, which one(s) are best?

1. If you were told some fuels are better for your health than others, would you be willing to change?

| Yes | No | Don’t know |
| --- | --- | --- |

1. a)If the best fuel for your health was more expensive than the one you currently use, would you be willing to change?

| Yes | No | Don’t know |
| --- | --- | --- |

BIRR

b)- How much per week extra would you be ready to pay?

1. If I tell you that cooking smoke is bad for your health:

a)-Would you be willing to do something about it?

| Yes | No | Don’t know |
| --- | --- | --- |

- if yes, explain:

b)-Would you be willing to cook outside?

| Yes | No | Don’t know |
| --- | --- | --- |

c)-Would you be willing to cook in a separate area?

| Yes | No | Don’t know |
| --- | --- | --- |

d)-Would you be willing to invest in a smoke free stove?

| Yes | No | Don’t know |
| --- | --- | --- |

BIRR

-If yes, how much money would you be willing to spend?

e) would you accept a smoke free stove if it was free of charge?
